# Characterization of microbial community structure during continuous anaerobic digestion of straw and cow manure

**DOI:** 10.1111/1751-7915.12298

**Published:** 2015-07-08

**Authors:** Li Sun, Phillip B Pope, Vincent G H Eijsink, Anna Schnürer

**Affiliations:** 1 Department of Microbiology, Swedish University of Agricultural Science, Uppsala BioCenterP.O. Box 7025, SE-750 07, Uppsala, Sweden; 2 Department of Chemistry, Biotechnology and Food Science, Norwegian University of Life SciencesP.O. Box 5003, NO-1432, Ås, Norway

## Abstract

Responses of bacterial and archaeal communities to the addition of straw during anaerobic digestion of manure at different temperatures (37°C, 44°C and 52°C) were investigated using five laboratory-scale semi-continuous stirred tank reactors. The results revealed that including straw as co-substrate decreased the species richness for bacteria, whereas increasing the operating temperature decreased the species richness for both archaea and bacteria, and also the evenness of the bacteria. Taxonomic classifications of the archaeal community showed that *Methanobrevibacter* dominated in the manure samples, while *Methanosarcina* dominated in all digesters regardless of substrate. Increase of the operating temperature to 52°C led to increased relative abundance of *Methanoculleus* and *Methanobacterium*. Among the bacteria, the phyla Firmicutes and Bacteroidetes dominated within all samples. Compared with manure itself, digestion of manure resulted in a higher abundance of an uncultured class WWE1 and lower abundance of Bacilli. Adding straw to the digesters increased the level of Bacteroidia, while increasing the operating temperature decreased the level of this class and instead increased the relative abundance of an uncultured genus affiliated to order MBA08 (Clostridia). A considerable fraction of bacterial sequences could not be allocated to genus level, indicating that novel phylotypes are resident in these communities.

## Introduction

In recent years, the search for renewable energy resources to replace fossil fuels has received increasing attention worldwide. Anaerobic digestion (AD) represents a highly interesting approach in this regard, since it allows various organic waste materials and dedicated energy crops to be converted to a renewable energy carrier (biogas), and produces a nutrient-rich residue that can be used as fertilizer in agriculture (Weiland, 2010; Appels *et al*., 2011; Nkoa, 2014). Among various possible substrates, agricultural residues, such as manure and straw, offer great potential for AD (Wang *et al*., 2009; Appels *et al*., 2011; Tsavkelova and Netrusov, 2012). However, the use of these materials for biogas production is still somewhat restricted because of their high content of lignocellulose, which is difficult to degrade due to its intricate structure, with cellulose fibres tightly linked to hemicellulose and lignin (Tsavkelova and Netrusov, 2012). Consequently, lignocellulosic materials typically result in slow degradation and low biogas yields (Angelidaki and Ellegaard, 2003; Hendriks and Zeeman, 2009; Frigon and Guiot, 2010). Moreover, using straw for biogas production requires co-digestion with a nutrient-rich material, as straw has a high C/N ratio and low levels of trace metals (Lehtomäki *et al*., 2007; Estevez *et al*., 2012; Risberg *et al*., 2013).

The biogas potential of straw can be improved by a combination of different measures, but so far no clear and generally applicable solution has been identified. One way to improve the digestibility is to use pretreatment techniques, such as steam explosion, but such strategies increase energy consumption, possibly threatening the economic feasibility of the process (Hendriks and Zeeman, 2009; Frigon and Guiot, 2010; Galbe and Zacchi, 2012). Some attempts have also been made to improve the degradation efficiency by using hydrolytic enzymes, as a pretreatment or direct additive, or by addition of specific cellulose-degrading bacteria (Parawira, 2012; Peng *et al*., 2014). Although not providing clear answers yet, these studies clearly indicate the potential for improved degradation efficiency through increased knowledge of cellulose-degrading organisms, and eventually more efficient cellulose conversion.

The formation of methane proceeds via a complex process involving four microbial steps: hydrolysis, fermentation, acetogenesis and methanogenesis (Zinder and Koch, 1984; Angelidaki *et al*., 2011). When lignocellulose-rich materials, such as straw, are used as substrate for biogas production, hydrolysis of cellulose was suggested to be the rate-limiting step (Noike *et al*., 1985; Lynd *et al*., 2002). The taxonomy and phylogeny of microbial communities within the AD process have been studied by various culture-based and molecular methods, including construction of clone libraries and sequencing by targeting 16S rDNA and functional genes (Klocke *et al*., 2008; Liu *et al*., 2009; Sun *et al*., 2013; Vanwonterghem *et al*., 2014; Ziganshina *et al*., 2014). In addition, the development and application of next-generation sequencing technologies has enabled time and cost-efficient studies of the microbial communities in various biogas processes (Werner *et al*., 2011; Sundberg *et al*., 2013; Ziganshin *et al*., 2013; Yang *et al*., 2014). The microorganisms involved in the hydrolysis step have been studied in ruminating animals, and more recently also in the biogas process. In the rumen of cattle, ubiquitous detected genera include *Clostridium*, *Bacteroides*, *Succinivibrio*, *Prevotella* and *Ruminococcus* (Dowd *et al*., 2008; Callaway *et al*., 2010). *Fibrobacter*, formerly grouped to *Bacteroides*, as well as *Ruminococcus* and uncultured bacteria have also been suggested to play important roles in cellulose hydrolysis in the rumen (Leschine, 1995; Ransom-Jones *et al*., 2012). For the biogas production process, just a few previous studies have specifically addressed the hydrolysis step. In a recent study by Lebuhn and colleagues (2014), Firmicutes and Bacteroidetes were suggested to be important in a hydrolytic/acidogenic digester fed with dried hay and straw. These two phyla have also been shown to be important during batch digestion of wheat straw and swine manure (Li *et al*., 2014).

In a previous study, we investigated the cellulolytic community in biogas digesters operating at different temperatures with a mixture of straw and manure or manure alone, by specifically targeting the glycoside hydrolase families 5 and 48 (Sun *et al*., 2013). In that study, all sequenced clones belonged to the phyla Firmicutes and Bacteroidetes. However, the entire microbial community was not investigated, and the total response to the addition of straw and to the temperature shifts remains unclear.

To gain a better understanding of the response of the microbial community to straw-rich materials, in the present study the same laboratory-scale semi-continuous reactors were employed for extended analysis of both the bacterial and archaeal communities by means of 454-pyrosequencing. More specifically, the aims of this study were to examine (i) the microbial communities using manure as sole substrate, (ii) the microbial response caused by addition of straw and (iii) the impact of different operating temperatures.

## Results

### Ecological index of archaeal and bacterial community

**Figure 1:**
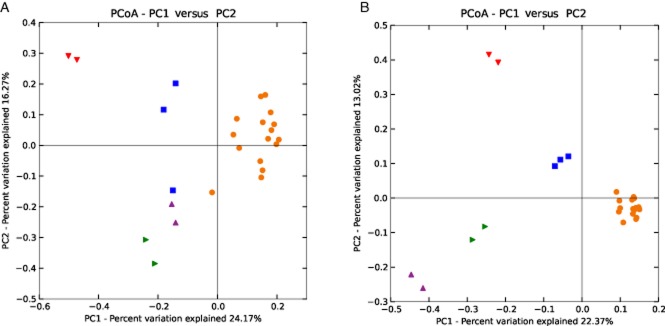
(A) Archaeal and (B) bacterial phylogenetic distances between samples as determined by unweighted UniFrac principal coordinate analysis (red = manure; blue = digester RM; orange = digester R^Tc^SS and R^37^SS; green = digester R^44^SS; purple = digester R^52^SS).

Amplicon pyrosequencing of 25 samples yielded 77 791 and 64 731 non-chimeric reads for archaea and bacteria respectively. The number of operational taxonomic units (OTUs) per sample ranged from 12 to 25 for archaea and from 112 to 277 for bacteria (Table 1). The phylogenetic compositions as determined by principal coordinate analysis (PCoA) of unweighted UniFrac matrices (Fig. 1) for the archaeal and bacterial communities within the same digester at multiple sampling points over time were similar. For instance, the four sampling points of the parallel digesters R^Tc^SS (reactors operated with steam-exploded straw and manure at 37°C, Risberg *et al*., 2013) were clustered closely together, indicating comparable phylogenetic structures within a total period of 91 days. Moreover, the communities in all parallel digesters running under the same conditions were also comparable, e.g. the community in duplicate reactors running with steam-exploded straw and manure at different temperatures (R^37^SS, R^44^SS and R^52^SS) was also similar at individual time points. The estimated richness for all samples analysed at all sampling points based on the Chao1 index indicated that the observed phylotypes covered 23–93% and 48–82% of the archaeal and bacterial populations respectively (Table 1). In general, the species richness expressed as the number of observed OTUs decreased as the operating temperature increased for both bacteria and archaea (Table 1 and Fig. S1). For bacterial reads, no species richness difference was observed between manure itself and the digester operated with manure alone (RM). However, a slightly lower bacterial species richness was observed in digesters that received straw in the substrate (digesters R^Tc^SS and R^37^SS) than that operated with manure alone (RM) (Welch’s *t-*test, *P* < 0.01). In contrast, for archaea, no clear trend in species richness was observed when comparing the manure itself and the mesophilic digesters operating with manure, alone or combined with straw. The Simpson diversity index ranged from 0.53 to 0.78 for archaea and from 0.72 to 0.98 for bacteria (Table 1). For the bacterial community, a lower Simpson index was observed during the increase in operating temperature, suggesting lower community evenness in these digesters (R^44^SS and R^52^SS, digesters processing straw and manure at 44°C and 52°C). Compared with the bacterial community, the Simpson index was generally lower for the archaeal community, and with the lowest value in R^44^SS compared with R^37^SS and R^52^SS (Welch’s *t-*test, *P* < 0.05). The Shannon diversity index varied from 1.46 to 2.64 for archaea and from 3.45 to 6.36 for bacteria (Table 1). Within the bacterial community, the Shannon index was comparably lower in digesters operating with straw at higher temperatures, i.e. at 44°C and 52°C compared with 37°C (Welch’s *t-*test, *P* < 0.05). For the archaeal community, a lower Shannon diversity index was observed in R^44^SS compared with R^37/Tc^SS and R^52^SS (Welch’s *t-*test, *P* < 0.05), and in RM compared with R^37/Tc^SS (Welch’s *t-*test, *P* < 0.01).

**Table 1:** Summary of observed OTUs, Chao1, Shannon and Simpson index in manure (S1 and S2 correspond to duplicate samples) and in laboratory-scale digesters processing: (1) manure as sole substrate (RM); (2) stream-exploded straw and manure, operating constantly at 37°C (R^Tc^SS); and (3) steam-exploded straw and manure, operating temperatures 37°C, 44°C and 52°C (R^37^SS, R^44^SS and R^52^SS), where R1 and R2 represent parallel digesters

Sample	Archaea				Bacteria			
	Chao1	OTUs	Shannon	Simpson	Chao1	OTUs	Shannon	Simpson
Manure S1	22	20	2.081	0.648	358	270	6.361	0.971
Manure S2	27	25	2.404	0.693	420	274	6.167	0.957
RM Day 42	69	23	2.028	0.639	487	266	6.355	0.975
RM Day 85	18	13	1.848	0.607	553	277	6.292	0.972
RM Day 148	22	18	1.870	0.597	440	253	5.819	0.956
R^Tc^SS R1 Day 9	26	18	2.369	0.747	320	191	4.896	0.910
R^Tc^SS R2 Day 9	26	20	2.367	0.723	371	225	5.550	0.949
R^37^SS R1 Day 9	20	17	2.271	0.736	398	209	5.221	0.932
R^37^SS R2 Day 9	28	22	2.309	0.714	425	203	5.197	0.934
R^Tc^SS R1 Day 38	23	20	2.643	0.777	309	197	5.463	0.952
R^Tc^SS R2 Day 38	47	24	2.612	0.759	319	198	5.524	0.953
R^37^SS R1 Day 38	22	20	2.295	0.737	325	212	5.377	0.936
R^37^SS R2 Day 38	28	23	2.117	0.632	398	209	5.494	0.951
R^Tc^SS R1 Day 80	55	20	2.390	0.744	421	244	6.134	0.969
R^Tc^SS R2 Day 80	26	21	2.237	0.682	315	217	5.811	0.959
R^37^SS R1 Day 80	21	18	1.740	0.533	374	229	5.688	0.950
R^37^SS R2 Day 80	101	23	2.438	0.751	358	234	5.819	0.948
R^Tc^SS R1 Day 99	21	18	2.000	0.639	375	235	5.988	0.965
R^Tc^SS R2 Day 99	44	16	1.704	0.569	334	221	5.900	0.959
R^37^SS R1 Day 99	32	22	2.530	0.736	321	232	6.064	0.968
R^37^SS R2 Day 99	78	23	2.399	0.729	391	224	5.827	0.959
R^44^SS R1 Day 224	21	14	1.618	0.594	191	156	5.015	0.923
R^44^SS R2 Day 224	22	15	1.456	0.545	255	158	4.906	0.926
R^52^SS R1 Day 402	13	12	2.150	0.718	149	112	3.446	0.717
R^52^SS R2 Day 402	17	15	2.233	0.731	165	119	4.023	0.787

### Comparative analysis of archaeal communities across samples

**Figure 2:**
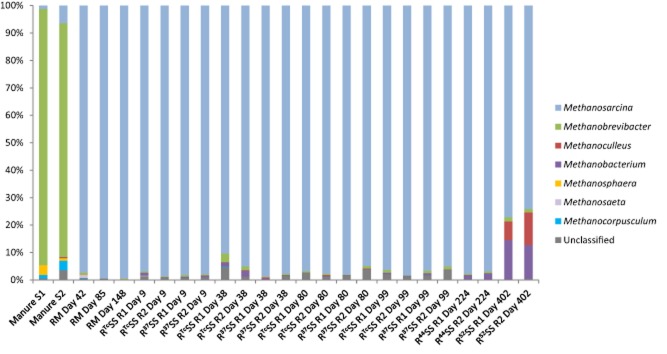
Relative abundance of archaea 16S rRNA gene at the genus level in manure (S1 and S2 correspond to duplicate samples) and in laboratory-scale digesters processing: (1) manure as sole substrate (RM); (2) stream-exploded straw and manure, operating constantly at 37°C (R^Tc^SS); and (3) steam-exploded straw and manure, operating temperatures 37°C, 44°C and 52°C (R^37^SS, R^44^SS and R^52^SS), where R1 and R2 represent parallel digesters.

For archaea, the OTU table was randomly subsampled 640 times prior to downstream analysis. It was possible to assign more than 99.6% of the sequences at the phylum level, among which more than 98.1% belonged to the phylum Euryarchaeota (mainly the classes Methanomicrobia and Methanobacteria). The rest of the sequences (< 1.9%) were represented by Crenarchaeota and unclassified sequences. For each sample, at least 95.1% of the sequences could be assigned to genus level (Fig. 2). *Methanosarcina* from the order Methanosarcinales, known to utilize both acetate and hydrogen, was the most dominant genus in digesters RM (97.4–99.5%), R^Tc^SS and R^37^SS (90.3–98.8%), R^44^SS (96.9–97.7%), and R^52^SS (74.1–77.1%), while this genus represented only 1.3% and 6.4% of the reads from the two manure samples. Sequences belonging to the acetoclastic methanogen, *Methanosaeta*, were only detected in two samples and at a low level, in RM on day 42 (1.1%) and in R^Tc^SS R1 on day 9 (0.2%). For hydrogenotrophic methanogens, the genus *Methanoculleus* represented less than 0.3% in all digesters operating at 37°C and 44°C, but corresponded to 6.9–11.9% in R^52^SS. In the manure samples, the genus *Methanoculleus* represented less than 0.4% of the community; instead, the archaeal community was dominated by *Methanobrevibacter* from the order Methanobacteriales (85.2% and 93.3%). However, the levels of *Methanobrevibacter* were low in all digesters (< 3.1%). In addition, sequences related to the genus *Methanobacterium* were found at low levels in the low-medium temperature digesters (37–44°C) and in the manure (< 2.5%), but were more abundant at the higher digestion temperature (12.3–14.4% in R^52^SS).

### Comparative analysis of bacterial communities across samples

The bacterial OTU table was rarefied 1700 times according to the sample having the lowest sequence counts. It was possible to assign at least 97.2% of the sequence reads at the phylum level. However, only 10.7–38.8% of total sequences could be allocated to known genus (Fig. S2). Firmicutes, Bacteroidetes and Cloacimonetes were identified as the three most dominant phyla within the bacterial community (Fig. S3). In addition, sequences belonging to Acidobacteria, Actinobacteria, Chloroflexi, Fibrobacteres, Planctomycetes, Proteobacteria, Synergistetes, Tenericutes, Thermi and Verrucomicrobia were detected in some of the samples, but at low occurrence (below 3.3% for each sample).

**Figure 3:**
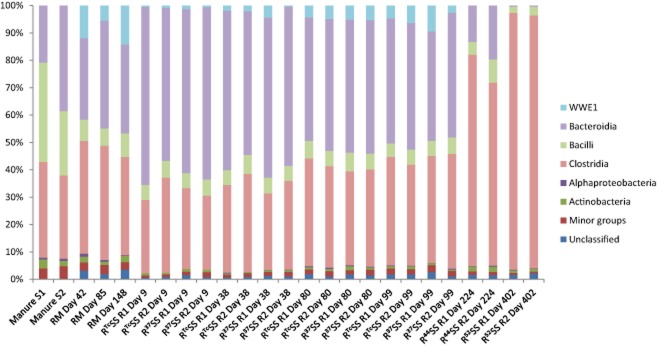
Relative abundance of bacterial 16S rRNA gene at the class level in manure (S1 and S2 correspond to duplicate samples) and in laboratory-scale digesters processing: (1) manure as sole substrate (RM); (2) stream-exploded straw and manure, operating constantly at 37°C (R^Tc^SS); and (3) steam-exploded straw and manure, operating temperatures 37°C, 44°C and 52°C (R^37^SS, R^44^SS and R^52^SS), where R1 and R2 represent parallel digesters.

**Figure 4:**
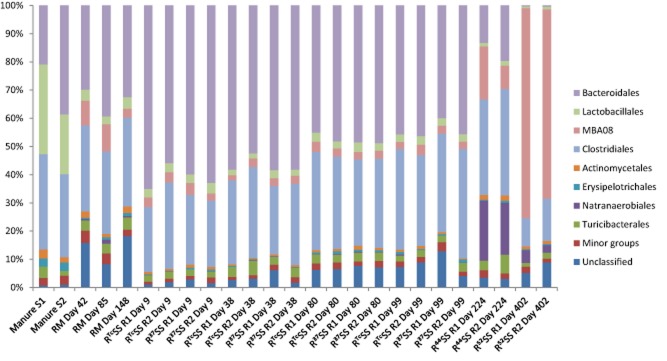
Relative abundance of bacterial 16S rRNA gene at the order level in manure (S1 and S2 correspond to duplicate samples) and in laboratory-scale digesters processing: (1) manure as sole substrate (RM); (2) stream-exploded straw and manure, operating constantly at 37°C (RTcSS); and (3) steam-exploded straw and manure, operating temperatures 37°C, 44°C and 52°C (R37SS, R44SS and R52SS), where R1 and R2 represent parallel digesters.

Firmicutes was observed in all digesters as well as in the manure samples, with the highest relative abundance in the digesters operated at 52°C (97.6–97.8%), followed by the digesters operated at 44°C (76.5–82.5%). In the remaining samples, the relative abundance of sequences belonging to this phylum was 56.8–74.1% (manure), 45.9–50.4% (RM, digester operated with manure alone) and 32.6–49.5% (R^37^SS and R^Tc^SS, Fig. S3). Within the Firmicutes, sequences belonging to the classes Bacilli and Clostridia dominated (Fig. 3). The class Clostridia was represented in similar levels in the digesters operated at 37°C (26.8–41.9%) and in the manure samples (30.4–34.9%). However, in line with the increase in operating temperature, the relative abundance of sequences within this class increased and was 66.8–77.2% and 92.4–93.8% in R^44^SS and R^52^SS respectively. Within the class Clostridia, sequences belonging to the order Clostridiales and to the uncultured order MBA08 were found to be dominant (Fig. 4). Clostridiales was present in manure (29.4–33.7%) and all digesters (10.1–38.8) with the lowest relative abundance at 52°C (10.1–15.0%), while MBA08 was not detected in manure but in all digesters (1.9–74.4%) with the highest relative abundance at 52°C (67.1–74.4%). Within the order Clostridiales, the families Peptostreptococcaceae (5.4–18.1%) and Clostridiaceae (2.2–11.1%) were identified as the major groups. In addition, Lachnospiraceae, Ruminococcaceae, Syntrophomonadaceae, Peptococcaceae, Catabacteriaceae, Eubacteriaceae and Veillonellaceae were present at lower levels (data not shown). Within the Clostridiaceae, sequences belonging to the genera *Clostridium* and *Sedimentibacter* dominated (Fig. S2). *Clostridium* was detected in all sampling points (0.7–4.4%). *Sedimentibacter* was absent in R^52^SS but detected in the other samples, with a slightly higher level in digesters receiving straw (3.0–6.9%). For the class Bacilli, a higher relative abundance was seen in the manure samples (23.5–36.2%) than in all digester samples (2.3–8.6%, Fig. 3). The orders Lactobacillales and Turicibacterales represented the major fraction of sequences within this class in all samples (Fig. 4). Lactobacillales had a higher relative abundance in manure samples compared with digester samples.

For the phylum Bacteroidetes, the class Bacteroidia represented more than 98.8% of the sequences in all samples (Fig. 3). However, at the genus level, most of the sequences within this phylum were represented by uncultured clones. The relative level of the phylum Bacteroidetes in the digester operating with manure was similar to that in the raw manure (21.0–39.4%), but lower than that in the digesters co-digesting straw at 37°C (40.0–65.1%). In contrast to the response to straw addition, the relative abundance of Bacteroidetes decreased with increased operating temperature. At 44°C and 52°C, Bacteroidetes represented 13.2–16.6% and 0.40% of the population respectively (Fig. S3).

The candidate phylum Cloacimonetes was present at levels corresponding to less than 0.2% in the manure samples, but at higher levels in digesters operating with only manure (6.0–14.5%) (Fig. S3). In the digesters operating with straw, the level of this phylum varied from 0.30% to 10.3% during operation at 37°C, and it was not detected at all in digesters operated at 44°C and 52°C. Within all the digesters operated at 37°C, sequences belonging to the uncultured cluster WWE1 (at class level) contributed to the majority of this phylum (Fig. 3).

## Discussion

The sequence analysis showed that the microbial community structure in digesters with the same treatment and in parallel digesters was relatively stable and similar over the whole operating period. This stability indicates that the differences in communities observed for different groups of digesters in the present study were caused by different operating conditions (i.e. temperature, substrate) and not just by variations due to time.

For archaea, the dominant phylum was Euryarchaeota, as found previously in other pyrosequencing studies on anaerobic digesters (Cardinali-Rezende *et al*., 2009; Riviere *et al*., 2009; Ritari *et al*., 2012; Zakrzewski *et al*., 2012). For bacteria, Firmicutes and Bacteroidetes have been repeatedly identified as the main phyla in various anaerobic digesters (Klocke *et al*., 2007; Garcia *et al*., 2011; Jaenicke *et al*., 2011; Kampmann *et al*., 2012; St-Pierre and Wright, 2014; Lucas *et al*., 2015). The presence of large proportions of unknown sequences at the genus level, as observed here, has also been reported in other amplicon sequencing studies (Zakrzewski *et al*., 2012; Lu *et al*., 2013; Li *et al*., 2014; Smith *et al*., 2014), as well as during construction of the a 16S rDNA clone library (Liu *et al*., 2009). It is clear that isolation and cultivation of uncultured microorganisms would provide deeper insights into their function in the AD process, as suggested previously (Narihiro and Sekiguchi, 2007; Curtis *et al*., 2013).

### Manure versus mesophilic AD of manure

Manure can be used as the sole substrate during AD, but typically results in low biogas yields (Mata-Alvarez *et al*., 2000; Angelidaki and Ellegaard, 2003). Co-digestion with more energy-rich materials can result in higher yields. On the other hand, the presence of manure can be a precondition for enabling AD of materials with a high C/N ratio and low buffering capacity, such as straw (Mata-Alvarez *et al*., 2014). Manure contributes nitrogen, trace elements and buffering components, and thus creates more suitable conditions for the growth of microbes, including methanogens, in the AD process (Mata-Alvarez *et al*., 2000). Possibly, effects by manure could also be caused by microbial inoculation. It is well known that the rumen has high cellulose-degrading efficiency and the bacteria involved in this may also be involved in degradation in the AD process. Methanogens active in the rumen may also contribute to methanogenesis in the biogas production process. In our diversity index analysis of the archaeal and bacterial communities, no difference was observed between the manure and the digester operated with manure (Table 1). However, community composition analysis revealed a clear difference between the digester and the manure for both archaea and bacteria (Figs 4). In the manure samples, *Methanobrevibacter* from the order Methanobacteriales was the most dominant archaeal genus (85.2% and 93.3%), showing similarity to the methanogenic community structure in the rumen of cows (Janssen and Kirs, 2008; Danielsson *et al*., 2012). However, when the manure was used as substrate for biogas production, the number of sequences affiliated to this hydrogenotrophic genus represented less than 3.2% and instead the *Methanosarcina* became dominant (> 97%). The cause of the decrease in relative abundance of *Methanobrevibacter* is not clear. The dominance of *Methanosarcina* is, however, in line with previous studies of manure-based digesters (Karakashev *et al*., 2005; Demirel and Scherer, 2008; Goberna *et al*., 2010; St-Pierre and Wright, 2014). High abundance of this methanogen has been suggested to be caused by its relatively high growth rate and ability to tolerate conditions inhibitory to other methanogens, such as presence of ammonia (De Vrieze *et al*., 2012). However, the digesters investigated in this study had low levels of ammonia, and similar to the manure (Risberg *et al*., 2013), thus this parameter is not the likely cause for the shift in the methanogenic community. *Methanosarcina* can also use the hydrogenotrophic pathway for methanogenesis, unlike the other acetotrophic methanogen *Methanosaeta*, and this might also enhance its competitive ability, even at low acetate levels as prevailing in the investigated digesters (De Vrieze *et al*., 2012). Another possible explanation for the dominance of this methanogen is the once-per-day feeding regime, which has been reported before to select for *Methanosarcina* rather than *Methanosaeta* (Conklin *et al*., 2006).

Analysis of the bacterial community identified Bacteroidetes (21.0–39.4%) and Firmicutes (45.9–74.1%) as the major phyla in both the manure samples and the digesters operating with manure (Fig. S3). This confirms previous findings on bacterial populations in the rumen of cows (Kim *et al*., 2011; Danielsson *et al*., 2012), manure digesters (Kampmann *et al*., 2012; St-Pierre and Wright, 2014) and other biogas digesters (Krause *et al*., 2008; Schlüter *et al*., 2008; Lee *et al*., 2012; Sundberg *et al*., 2013; Ziganshin *et al*., 2013; Li *et al*., 2014). However, as for the archaeal population, statistical analysis revealed that the bacterial community in the digester was separated from the manure sample (Fig. 1B). This was most likely caused by the substantially higher amount (6.0–14.5%) of sequences related to the candidate phylum Cloacimonetes in the manure digesters than in the manure itself. Representatives of this phylum seem not to be specific for manure as they have been detected also in digesters operating with other substrates (Cardinali-Rezende *et al*., 2009; Sundberg *et al*., 2013; Li *et al*., 2014) and in the rumen (Piao *et al*., 2014). In the present study, sequences belonging to the uncultured WWE1 contributed to the majority of the candidate phylum Cloacimonetes (Fig. 3). This uncultured cluster was first discovered as a subdominant group (up to 10%) in an anaerobic sludge digester (Chouari *et al*., 2005), but later also in other anaerobic digesters (Riviere *et al*., 2009; Lucas *et al*., 2015). The genome of one species from this phylum, namely ‘*Candidatus* Cloacamonas acidaminovorans’, was recovered from a metagenomic study, which suggested it as a syntrophic bacterium capable of degrading propionate and amino acids (Pelletier *et al*., 2008). In a recent study, evidence emerged suggesting that this group of bacteria also is involved in the AD of cellulose, through an extracellular cellulose hydrolysis process and/or in the fermentation of organic substrates originating from cellulose (Limam *et al*., 2014). Variation in the bacterial community between the manure and the digester was also seen at different taxonomic levels, with the most pronounced difference within the phylum Firmicutes. Here, a significantly higher relative abundance of sequences belonging to the order of Lactobacillales (from class Bacilli) was observed in the manure samples than in the manure digester. The relative abundance of Clostridia was rather similar in all digesters, which resulted in a higher Clostridia/Bacilli ratio in the manure digester than in the manure itself. A higher level of Clostridia in relation to Bacilli was also seen in a previous study analysing three different manure digesters (St-Pierre and Wright, 2014). The cause of the increase in Clostridia/Bacilli ratio in the digesters relative to the manure is not clear, but one possible explanation is that the availability of easily fermentable sugar is comparably lower in the anaerobic digester (Walter *et al*., 2011).

### Microbial response caused by straw

As described in our previous paper, adding straw (steam-exploded or non-pretreated) as a co-substrate during the digestion of manure did not impact on the overall process stability or performance (gas yield and degree of degradation, Risberg *et al*., 2013); however, a response was obtained for the microbial community. For archaea, no difference was observed for the major population during operation at 37°C with manure as the sole substrate or with a mixture of manure and straw. Addition of straw had a more pronounced effect on the bacterial community, for example reflected in a significant decrease in bacterial species richness compared with digestion of manure alone (Table 1 and Fig. S1). A similar decrease in species richness was observed in a study by Li and colleagues (2014), where addition of straw resulted in a decrease in the number of observed species and in the Shannon index. This result possibly reflects selection and enrichment of specialists involved in degradation of the straw. At the phylum level, the addition of straw resulted in an increase in the relative abundance of Bacteroidetes compared with the digesters processing manure alone. However, sequences affiliated with Firmicutes were at similar abundance in all digesters operated at 37°C. This result was somewhat surprising, as Firmicutes contains many isolated cellulose degraders (Lynd *et al*., 2002), and several studies in biogas digesters have suggested representatives of this phylum, particularly belonging to the order Clostridiales, to be key players for degradation of cellulose (Krause *et al*., 2008; Hanreich *et al*., 2013; Li *et al*., 2013; Lu *et al*., 2013; Lebuhn *et al*., 2014). However, in the present study, a higher abundance of *Sedimentibacter* was observed in the digesters operating with straw, in similarity to a study by Li and colleagues (2014). However, this is not necessarily an expected change, as the two species isolated and characterized from this genus so far are not capable of utilizing carbohydrates (Breitenstein *et al*., 2002).

In line with our results, a recent metagenomic study also found an increase in the level of Bacteroidetes in anaerobic batch fermentation of straw and hay (Hanreich *et al*., 2013). In this study, the number of expressed sugar transporters increased simultaneously with the level of Bacteroidetes, which was assumed to be responsible for degradation of polysaccharides. Representatives of the phylum Bacteroidetes have been shown to have the capacity to degrade a wide variety of plant polysaccharides, including cellulose (Robert *et al*., 2007; Hatamoto *et al*., 2014; Naas *et al*., 2014). However, recent studies show that cellulose conversion in Bacteroidetes needs further investigation (Naas *et al*., 2014), and thus the role of this phylum during straw degradation remains somewhat unclear. The straw in this study was pretreated by steam explosion (Risberg *et al*., 2013). This treatment disrupts the intricate structure of lignocellulose and releases more simple carbohydrates, possibly explaining the increased level of Bacteroidetes.

### Impact of temperature

For archaeal and bacterial communities, the species richness decreased in line with the increase in process temperature. Similarly, Sundberg and co-workers (2013) observed that among 21 full-scale biogas digesters, thermophilic co-digestion plants had lower microbial richness than mesophilic plants. A similar trend was also observed in a methanogenic digester processing organic household waste at 37°C and at 55°C, where a higher species richness was found for both the archaeal and bacterial community at the mesophilic temperature (Levén *et al*., 2007). Interestingly, the species richness of the archaea was lowest at 44°C, suggesting that an increase in temperature with only a few degrees from 37°C has an impact on the diversity of this community. This result was in line with a previous study where a significant decrease in species richness of methanogens was observed after an increase in operational temperature from 37°C to 42°C in lab-scale digesters processing municipal solid waste supplemented with albumin (Westerholm M., Isaksson S., Müller B., and Schnürer A., unpublished). Thus, it is apparent that temperature is a strong regulating factor for the microbial community in the biogas process, and an increase in temperature restricts the number of species present. Notably, the previously published operating data for these digesters show that this decrease in richness of both bacteria and archaea and in the evenness of bacteria did not affect the overall performance of the digesters (Risberg *et al*., 2013). Moreover, in the study by Westerholm *et al*. (unpublished), the increase in temperature to 42°C even resulted in increased methane yields compared with operation at 37°C, in spite of a decreased species richness of methanogens.

For the archaeal community, after an increase in operating temperature from 37°C to 52°C, there was an increase in the relative abundance of the genera *Methanobacterium* and *Methanoculleus*, from the order Methanobacteriales and Methanomicrobiales respectively. Similar results have been reported for a laboratory-scale biogas digester fed with only cattle manure during a temperature shift from 39°C to 55°C, where a decrease in relative abundance of *Methanosarcina* from 89% to 54% was accompanied by an increase of *Methanoculleus* spp. (Ziganshin *et al*., 2013). Moreover, a sharp increase in the relative abundance of hydrogenotrophic methanogens *Methanobacterium*, *Methanoculleus* and *Methanothermobacter* has been observed in biogas digesters co-digesting cattle excreta and olive mill wastes during a temperature shift from 37°C to 55°C, likely induced by an increase in H_2_ partial pressure (Goberna *et al*., 2010).

For the bacterial community, the relative abundance of Firmicutes increased during the transition to the thermophilic temperature. This increase was mainly caused by a higher abundance of sequences related to the uncultured order MBA08 within the class Clostridia. Interestingly, this order was represented by a single uncultured genus, which partially explained the decreased species richness and evenness of the bacterial community. The MBA08 cluster was first discovered in a thermophilic laboratory-scale digester processing municipal solid waste (Tang *et al*., 2004) and also later in a thermophilic garbage digester (Cheon *et al*., 2007). Thus, it seems reasonable to assume that this cluster contains thermophilic Clostridia. In our previous study using the same anaerobic digesters and using a functional gene approach for community analysis, we also found that the major response to the temperature rise was an increase in the relative abundance of organisms belonging to the class Clostridia (Sun *et al*., 2013). In contrast to Firmicutes, the relative abundance of Bacteroidetes decreased with the increase in operating temperature in the present study. This trend was also seen in our previous study (Sun *et al*., 2013) and in the study by Ziganshin and colleagues (2013), who observed a decrease in sequences related to this phylum during a temperature shift from 38°C to 55°C in digesters processing cattle manure and agricultural waste. Moreover, introduction of cellulose into a thermophilic sludge digester resulted in increased abundance of Firmicutes and decreased abundance of Bacteroidetes (Lu *et al*., 2013). In addition, a high ratio of Firmicutes/Bacteroidetes has been observed in different digesters operated at high temperature, e.g. in three hydrolytic/acidogenic horizontal tubular digesters processing dried hay and straw, operating at 38°C, 45°C and 55°C (Lebuhn *et al*., 2014), in two digesters treating organic household waste at 37°C and 55°C (Levén *et al*., 2007), and in a thermophilic reactor operating with poultry manure (Smith *et al*., 2014). Temperature clearly has a strong effect on the bacterial community in a biogas digester, and the high relative abundance of Clostridia suggested a competitive advantage for this class.

## Conclusions

Comparing sequencing results from the manure used as substrate and the digesters operated under various conditions revealed that the microbial communities in the digesters were likely shaped by the operating conditions. For archaea, the hydrogenotrophic methanogens present in the manure were outcompeted in the digester environment by *Methanosarcina*, which can utilize both hydrogen and acetate. For bacteria, the relative abundance of Bacilli was higher in manure, while an uncultured cluster WWE1 affiliated to the phylum Cloacimonetes was more abundant in the digester operated with manure alone. After including straw for co-digestion with manure, no major changes were observed for the archaeal community. However, for bacteria, the abundance of Bacteroidia increased, suggesting importance of this group. Both the archaeal and bacterial communities responded to increased operating temperatures. For archaea, although *Methanosarcina* was the most dominant genus in all digester samples, *Methanoculleus* and *Methanobacterium* were considerably more abundant at the higher temperatures. For bacteria, a decrease in the relative abundance of Bacteroidia was accompanied by an increase in Clostridia. A considerable fraction of bacterial reads could not be allocated to genus level, meaning that more efforts to isolate and characterize unknown dominant microbes in anaerobic digesters are needed. Such efforts could provide deeper insights into microbial function in the AD process.

## Experimental procedures

### Anaerobic digesters

Samples for microbial community structure analysis were taken from five laboratory-scale semi-continuous stirred tank reactors (Dolly, Belach Bioteknik, Stockholm, Sweden) run under different conditions (Risberg *et al*., 2013). In addition to the digester samples, two samples were taken from the manure used as substrate for the digesters. A detailed description of the digesters, the chemical composition of the manure and the process performance can be found in Risberg and colleagues (2013). In this study, the phase referred to as ‘second experimental period’ was used for the microbial analysis. In brief, five set-ups of digesters (designated RM, R^Tc^SS, R^37^SS, R^44^SS and R^52^SS) were investigated in this study; all but RM operated in duplicate. All digesters had the same hydraulic retention time (HRT) of 25 days, and the organic loading rate was maintained at around 2.8 g VS l^−l^ day^−l^. The inoculum used for starting up the AD process (R^Tc^SS and R^37^SS) originated from the digester R^Lc^SS in Risberg and colleagues (2013), which operated at similar conditions with another batch of manure for 83 days (full load). All the digesters were fed 6 days a week and had stable and similar performance: the pH was 7.5–8.1, the ammonium-nitrogen level was 1.5–2.0 g kg^−1^ wet weight (1.2 g kg^−1^ wet weight for manure itself), no accumulation of acids occurred during the whole operating period, and the specific methane yield was 0.13–0.17 l CH_4_ kg^−1^ VS (Risberg *et al*., 2013). Digester RM operated with cow manure as the sole substrate at 37°C, while two parallel digesters R^Tc^SS operated with cow manure supplemented with steam-exploded straw (78/22% on a VS basis) at 37°C during the whole operating period. Another two parallel digesters R^37^SS were fed with the same substrate and initially operated under the same conditions as R^Tc^SS at 37°C, but both digesters were then subjected to a temperature rise to 44°C and further to 52°C. After around three HRTs of operation at the corresponding temperature (i.e. 44°C and 52°C), the digesters were named R^44^SS and R^52^SS respectively. The total operating time of the digesters was 402 days.

### DNA extraction

For digesters R^Tc^SS and R^37^SS, samples were withdrawn on days 9, 38, 80 and 99 of operation. The starting point for this experiment was set to the day when the digesters had been in operation for three HRTs at full load. For digesters R^44^SS and R^52^SS, samples were taken after operation at the corresponding temperature (44°C and 52°C, respectively) for around three HRTs, i.e. on days 224 and 402. For digester RM, samples were taken on days 42, 85 and 148. A 20 ml sample was withdrawn from each digester and stored at −20°C until analysis. Total genomic DNA was prepared in triplicate using the FastDNA Spin Kit for Soil (MP Biomedicals, Santa Ana, CA, USA). Aliquots of 200 μl digester sludge were used for extraction following the manufacturer’s protocol, and 60 μl water was used in the final elution of DNA. The concentrations of extracted DNA were measured using the Quant-iT dsDNA BR Assay Kit (Invitrogen, Life Technologies Europe, Stockholm, Sweden).

### 454-pyrosequencing

Primers targeting the bacterial 16S rRNA gene integrated with 454 Life Sciences adaptors 8F (5′-CCT ATC CCC TGT GTG CCT TGG CAG TCT CAG CAA CAG CTA GAG TTT GAT CCT GG-3′) and 515R (5′-CCA TCT CAT CCC TGC GTG TCT CCG ACT CAG NNN NNN NNT TAC CGC GGC TGC T-3′) were used for amplification from genomic DNA by polymerase chain reaction (PCR) (Stanley *et al*., 2013). For archaea, the primers 340F (5′-CCT ATC CCC TGT GTG CCT TGG CAG TCT CAG CAA AAG CTC CCT AYG GGG YGC ASC AG-3′) and 1 000R (5′-CCA TCT CAT CCC TGC GTG TCT CCG ACT CAG NNN NNN NNG GCC ATG CAC YWC YTC TC-3′) were used for amplification (Gantner *et al*., 2011). For samples from RM, R^Tc^SS and R^37^SS, the PCR amplification was performed with iProof High-Fidelity DNA Polymerase (Bio-Rad, Hercules, CA, USA). For samples R^44^SS and R^52^SS, PCR amplification was conducted using Maxima Hot Start PCR Master Mix (Fermentas, Thermo Fisher Scientific, Hudson, NH, USA) for bacteria and Bio-Rad iQ™ Supermix (Bio-Rad) for archaea. Each PCR reaction contained 12.5 μl of corresponding reaction mix, 0.5 μM of each primer, 20 ng of DNA template and 9.5 μl of sterile water (25 μl final volume). The PCR programmes were as follows: initial denaturation at 98°C for 3 min, 30 cycles (35 cycles for archaea) of denaturation at 98°C for 10 s, annealing at 55°C (57°C for archaea) for 45 s and elongation at 72°C for 45 s, followed by a final extension step of 7 min at 72°C. The size and purity of amplicons were checked by electrophoresis on 2% agarose gel. Short, non-specific amplification products were removed with AMP beads (AMPure XP, Beckman Coulter Genomics, Danvers, MA, USA) using the manufacturer’s protocol but with a modified bead to DNA volume ratio of 0.7:1. The concentrations of purified products were measured using the Quant-iT dsDNA BR Assay Kit (Invitrogen). All PCR products were pooled in equal molar amounts and further checked on 2% agarose gel. The mixed pool of PCR products from manure samples, RM, R^Tc^SS and R^37^SS, was sequenced at Norwegian Sequencing Centre in Oslo, while the one from R^44^SS and R^52^SS was sequenced at the Swedish Institute for Infectious Disease Control in Solna, using the Roche/454 GS Titanium technology platform. Sequences have been deposited in the National Center for Biotechnology Information (NCBI) Sequence Read Archive under the accession number SRP049689.

### 16S rRNA gene sequence analysis

The 16S rRNA gene sequences were processed using the qiime pipeline (Caporaso *et al*., 2010a). In summary, the dataset was first quality trimmed by removing sequences that were less than 400 or more than 600 bp in length, contained ambiguous bases, had a mean quality score < 25, contained a homopolymer run exceeding 6 bp, or did not contain a primer or barcode sequence. The usearch quality filter was used to remove chimera sequences (Edgar, 2010). The OTUs were determined using uclust at a threshold of 97% (Edgar, 2010). Representative sequences were selected as the most abundant sequence in each OTU and further aligned against the Greengenes core set (gg_13_8) using pynast software (Caporaso *et al*., 2010b; McDonald *et al*., 2012) and a minimum identity of 75%. Taxonomy was assigned to each OTU using the Ribosomal Database Project classifier (Wang *et al*., 2007) with a minimum confidence threshold of 80%. The alignment was filtered to remove gaps and hypervariable regions using a Lane mask, and a maximum-likelihood tree was constructed from the filtered alignment using FastTree (Price *et al*., 2010). The OTU tables were rarefied (according to the sample containing the smallest set of sequences) to equalize sampling depth and avoid heterogeneity (i.e. avoid bias from unequal sampling effort). From the OTU tables and phylogenetic trees, an unweighted UniFrac distance matrix was constructed and further visualized with PCoA (Lozupone and Knight, 2005). Rarefaction curve, observed species, chao1 (Hill *et al*., 2003), Shannon (Spellerberg and Fedor, 2003) and Simpson indices (Simpson, 1949) were computed by qiime alpha diversity analysis script (Caporaso *et al*., 2010).

## Supporting Information

**Fig. S1.** Rarefaction analysis of bacteria for manure (S1 and S2 correspond to duplicate samples, in purple) and for laboratory-scale digesters processing: (1) manure as sole substrate (RM, in blue); (2) stream-exploded straw and manure, operating constantly at 37°C (R^Tc^SS, in green); and (3) steam-exploded straw and manure, operating temperatures 37°C, 44°C and 52°C (R^37^SS, in green; R^44^SS, in yellow; and R^52^SS, in red), where R1 and R2 represent parallel digesters. The operational taxonomic units (OTUs) were determined at the sequence-similarity threshold of 97%. **Fig. S2.** Relative abundance of bacterial 16S rRNA gene at the genus level in manure (S1 and S2 correspond to duplicate samples) and in laboratory-scale digesters processing: (1) manure as sole substrate (RM); (2) stream-exploded straw and manure, operating constantly at 37°C (R^Tc^SS); and (3) steam-exploded straw and manure, operating temperatures 37°C, 44°C and 52°C (R^37^SS, R^44^SS and R^52^SS), where R1 and R2 represent parallel digesters. **Fig. S3.** Relative abundance of bacterial 16S rRNA gene at the phylum level in manure (S1 and S2 correspond to duplicate samples) and in laboratory-scale digesters processing: (1) manure as sole substrate (RM); (2) stream-exploded straw and manure, operating constantly at 37°C (R^Tc^SS); and (3) steam-exploded straw and manure, operating temperatures 37°C, 44°C and 52°C (R^37^SS, R^44^SS and R^52^SS), where R1 and R2 represent parallel digesters. **Table S1.** Summary of barcode sequences used for manure (S1 and S2 correspond to duplicate samples) and for laboratory-scale digesters processing: (1) manure as sole substrate (RM); (2) stream-exploded straw and manure, operating constantly at 37°C (R^Tc^SS); and (3) steam-exploded straw and manure, operating temperatures 37°C, 44°C and 52°C (R^37^SS, R^44^SS and R^52^SS), where R1 and R2 represent parallel digesters.
