# Supplementary material for: Characterization of microbial community structure during continuous anaerobic digestion of straw and cow manure
Source: Microb Biotechnol. 2015 Jul 8;8(5):815–27. doi: 10.1111/1751-7915.12298 (PMC4554469; doi:10.1111/1751-7915.12298)

Table S1. Summary of barcode sequences used for manure (S1 and S2 correspond to duplicate samples) and for laboratory-scale digesters processing: (1) manure as sole substrate (RM); (2) stream-exploded straw and manure, operating constantly at 37°C (R^Tc^SS); (3) steam-exploded straw and manure, operating temperature 37°C, 44°C and 52°C (R^37^SS, R^44^SS and R^52^SS), where R1 and R2 represent parallel digesters.

| Sample name | Archaea | |  | Bacteria | |
| --- | --- | --- | --- | --- | --- |
|  | 454 Sample ID | Barcode Sequence |  | 454 Sample ID | Barcode Sequence |
| Manure_S1 | 1000RTA21 | GGTTAAGG |  | 515RTB21 | AACGGCAA |
| Manure_S1 | 1000RTA43 | GTACGTTG |  | 515RTB43 | AAGGATCG |
| Manure_S1 | 1000RTA65 | GTCAACTG |  | 515RTB65 | ACACAGAG |
| Manure_S2 | 1000RTA22 | GGTTATCG |  | 515RTB22 | AACGGCTT |
| Manure_S2 | 1000RTA44 | GTACTCCT |  | 515RTB44 | AAGGATGC |
| Manure_S2 | 1000RTA66 | GTCAAGAG |  | 515RTB66 | ACACAGTC |
| RM_Day_42 | 1000RTA17 | GGTATAGG |  | 515RTB17 | AACGCCAT |
| RM_Day_42 | 1000RTA39 | GTACCTTC |  | 515RTB39 | AAGCTTCG |
| RM_Day_42 | 1000RTA61 | GTAGTCGT |  | 515RTB61 | AATTGCGC |
| RM_Day_85 | 1000RTA18 | GGTATTCG |  | 515RTB18 | AACGCCTA |
| RM_Day_85 | 1000RTA40 | GTACGAAG |  | 515RTB40 | AAGCTTGC |
| RM_Day_85 | 1000RTA62 | GTAGTGCT |  | 515RTB62 | AATTGGCC |
| RM_Day_148 | 1000RTA20 | GGTTAACC |  | 515RTB20 | AACGCGTT |
| RM_Day_148 | 1000RTA42 | GTACGTAC |  | 515RTB42 | AAGGAAGG |
| RM_Day_148 | 1000RTA64 | GTCAACAC |  | 515RTB64 | ACACACTG |
| RTCSS_R1_Day_9 | 1000RTA3 | GGCGATAA |  | 515RTB3 | AACCATCG |
| RTCSS_R1_Day_9 | 1000RTA25 | GGTTCCTT |  | 515RTB25 | AACGTTCG |
| RTCSS_R1_Day_9 | 1000RTA47 | GTACTGGT |  | 515RTB47 | AAGGCGAT |
| RTCSS_R1_Day_38 | 1000RTA7 | GGTAATGG |  | 515RTB7 | AACCGGAA |
| RTCSS_R1_Day_38 | 1000RTA29 | GGTTGCTA |  | 515RTB29 | AAGCATCC |
| RTCSS_R1_Day_38 | 1000RTA51 | GTAGAGGT |  | 515RTB51 | AAGGTTCC |
| RTCSS_R1_Day_80 | 1000RTA11 | GGTACGTT |  | 515RTB11 | AACCTTCC |
| RTCSS_R1_Day_80 | 1000RTA33 | GTACACGT |  | 515RTB33 | AAGCGCAA |
| RTCSS_R1_Day_80 | 1000RTA55 | GTAGCTTG |  | 515RTB55 | AATAGCGG |
| RTCSS_R1_Day_99 | 1000RTA15 | GGTAGGTA |  | 515RTB15 | AACGATCC |
| RTCSS_R1_Day_99 | 1000RTA37 | GTACCATG |  | 515RTB37 | AAGCTACC |
| RTCSS_R1_Day_99 | 1000RTA59 | GTAGGTTC |  | 515RTB59 | AATTCGGC |
| RTCSS_R2_Day_9 | 1000RTA4 | GGCGTATT |  | 515RTB4 | AACCATGC |
| RTCSS_R2_Day_9 | 1000RTA26 | GGTTCGAT |  | 515RTB26 | AACGTTGC |
| RTCSS_R2_Day_9 | 1000RTA48 | GTAGACCT |  | 515RTB48 | AAGGCGTA |
| RTCSS_R2_Day_38 | 1000RTA8 | GGTACCAT |  | 515RTB8 | AACCGGTT |
| RTCSS_R2_Day_38 | 1000RTA30 | GGTTGGAA |  | 515RTB30 | AAGCATGG |
| RTCSS_R2_Day_38 | 1000RTA52 | GTAGCAAG |  | 515RTB52 | AAGGTTGG |
| RTCSS_R2_Day_80 | 1000RTA12 | GGTAGCAA |  | 515RTB12 | AACCTTGG |
| RTCSS_R2_Day_80 | 1000RTA34 | GTACAGCT |  | 515RTB34 | AAGCGCTT |
| RTCSS_R2_Day_80 | 1000RTA56 | GTAGGAAC |  | 515RTB56 | AATAGGCG |
| RTCSS_R2_Day_99 | 1000RTA16 | GGTATACC |  | 515RTB16 | AACGATGG |
| RTCSS_R2_Day_99 | 1000RTA38 | GTACCTAG |  | 515RTB38 | AAGCTAGG |
| RTCSS_R2_Day_99 | 1000RTA60 | GTAGTCCA |  | 515RTB60 | AATTGCCG |
| R37SS_R1_Day_9 | 1000RTA1 | GGCCTTAA |  | 515RTB1 | AACCAACC |
| R37SS_R1_Day_9 | 1000RTA23 | GGTTATGC |  | 515RTB23 | AACGTACC |
| R37SS_R1_Day_9 | 1000RTA45 | GTACTCGA |  | 515RTB45 | AAGGCCAA |
| R37SS_R1_Day_38 | 1000RTA5 | GGCGTTAT |  | 515RTB5 | AACCGCAT |
| R37SS_R1_Day_38 | 1000RTA27 | GGTTCGTA |  | 515RTB27 | AAGCAACG |
| R37SS_R1_Day_38 | 1000RTA49 | GTAGACGA |  | 515RTB49 | AAGGTACG |
| R37SS_R1_Day_80 | 1000RTA9 | GGTACCTA |  | 515RTB9 | AACCTACG |
| R37SS_R1_Day_80 | 1000RTA31 | GGTTGGTT |  | 515RTB31 | AAGCCGAA |
| R37SS_R1_Day_80 | 1000RTA53 | GTAGCATC |  | 515RTB53 | AATACCGC |
| R37SS_R1_Day_99 | 1000RTA13 | GGTAGCTT |  | 515RTB13 | AACGAACG |
| R37SS_R1_Day_99 | 1000RTA35 | GTACAGGA |  | 515RTB35 | AAGCGGAT |
| R37SS_R1_Day_99 | 1000RTA57 | GTAGGATG |  | 515RTB57 | AATTCCGG |
| R37SS_R2_Day_9 | 1000RTA2 | GGCGAATA |  | 515RTB2 | AACCAAGG |
| R37SS_R2_Day_9 | 1000RTA24 | GGTTCCAA |  | 515RTB24 | AACGTAGG |
| R37SS_R2_Day_9 | 1000RTA46 | GTACTGCA |  | 515RTB46 | AAGGCCTT |
| R37SS_R2_Day_38 | 1000RTA6 | GGTAATCC |  | 515RTB6 | AACCGCTA |
| R37SS_R2_Day_38 | 1000RTA28 | GGTTGCAT |  | 515RTB28 | AAGCAAGC |
| R37SS_R2_Day_38 | 1000RTA50 | GTAGAGCA |  | 515RTB50 | AAGGTAGC |
| R37SS_R2_Day_80 | 1000RTA10 | GGTACGAA |  | 515RTB10 | AACCTAGC |
| R37SS_R2_Day_80 | 1000RTA32 | GTACACCA |  | 515RTB32 | AAGCCGTT |
| R37SS_R2_Day_80 | 1000RTA54 | GTAGCTAC |  | 515RTB54 | AATACGCC |
| R37SS_R2_Day_99 | 1000RTA14 | GGTAGGAT |  | 515RTB14 | AACGAAGC |
| R37SS_R2_Day_99 | 1000RTA36 | GTACCAAC |  | 515RTB36 | AAGCGGTA |
| R37SS_R2_Day_99 | 1000RTA58 | GTAGGTAG |  | 515RTB58 | AATTCGCG |
| R44SS_R1_Day_224 | 1000RTA204 | GGCGTATT |  | 515RTB204 | AACCATGC |
| R44SS_R1_Day_224 | 1000RTA205 | GGCGTTAT |  | 515RTB205 | AACCGCAT |
| R44SS_R1_Day_224 | 1000RTA206 | GGTAATCC |  | 515RTB206 | AACCGCTA |
| R44SS_R2_Day_224 | 1000RTA222 | GGTTATCG |  | 515RTB222 | AACGGCTT |
| R44SS_R2_Day_224 | 1000RTA223 | GGTTATGC |  | 515RTB223 | AACGTACC |
| R44SS_R2_Day_224 | 1000RTA224 | GGTTCCAA |  | 515RTB224 | AACGTAGG |
| R52SS_R1_Day_402 | 1000RTA213 | GGTAGCTT |  | 515RTB213 | AACGAACG |
| R52SS_R1_Day_402 | 1000RTA214 | GGTAGGAT |  | 515RTB214 | AACGAAGC |
| R52SS_R1_Day_402 | 1000RTA215 | GGTAGGTA |  | 515RTB215 | AACGATCC |
| R52SS_R2_Day_402 | 1000RTA231 | GGTTGGTT |  | 515RTB231 | AAGCCGAA |
| R52SS_R2_Day_402 | 1000RTA232 | GTACACCA |  | 515RTB232 | AAGCCGTT |
| R52SS_R2_Day_402 | 1000RTA233 | GTACACGT |  | 515RTB233 | AAGCGCAA |

Figure S1. Rarefaction analysis of bacteria for manure (S1 and S2 correspond to duplicate samples, in purple) and for laboratory-scale digesters processing: (1) manure as sole substrate (RM, in blue); (2) stream-exploded straw and manure, operating constantly at 37°C (R^Tc^SS, in green); (3) steam-exploded straw and manure, operating temperature 37°C, 44°C and 52°C (R^37^SS, in green; R^44^SS, in yellow; and R^52^SS, in red), where R1 and R2 represent parallel digesters. The operational taxonomic units (OTUs) were determined at the sequence-similarity threshold of 97%.


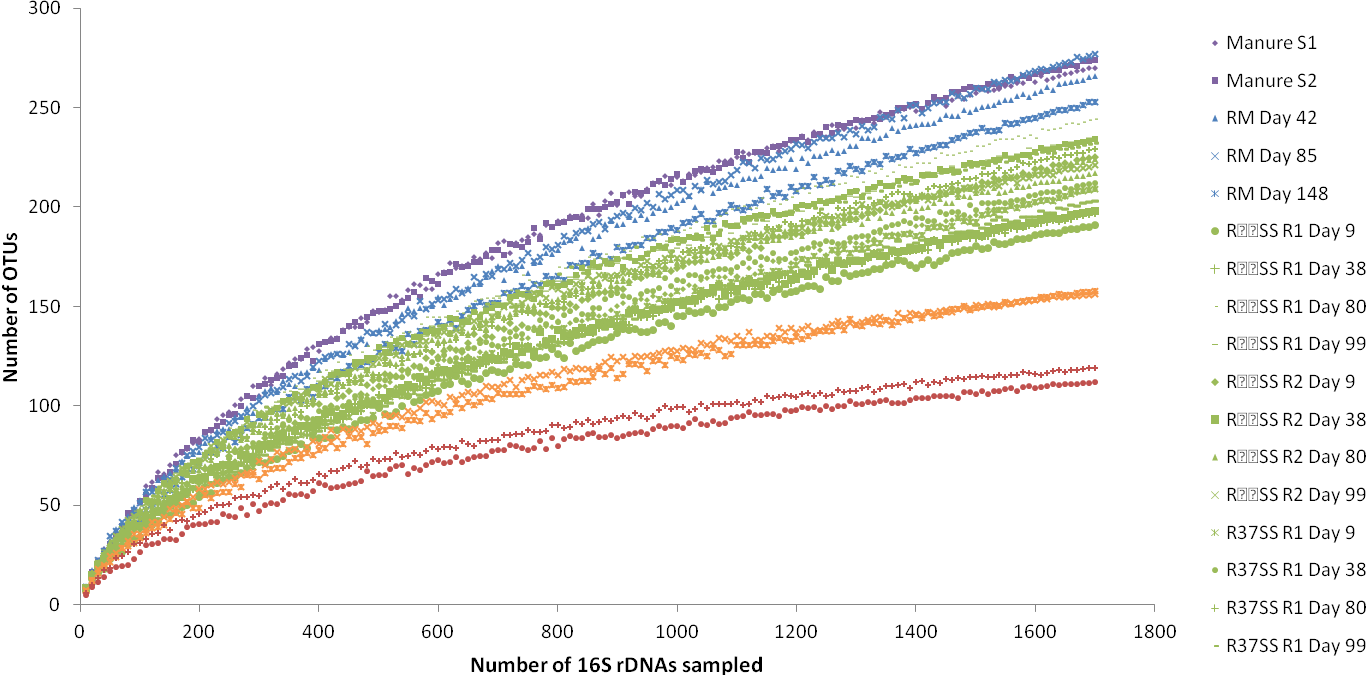


Figure S2. Relative abundance of bacterial 16S rRNA gene at genus level in manure (S1 and S2 correspond to duplicate samples) and in laboratory-scale digesters processing: (1) manure as sole substrate (RM); (2) stream-exploded straw and manure, operating constantly at 37°C (R^Tc^SS); (3) steam-exploded straw and manure, operating temperature 37°C, 44°C and 52°C (R^37^SS, R^44^SS and R^52^SS), where R1 and R2 represent parallel digesters.


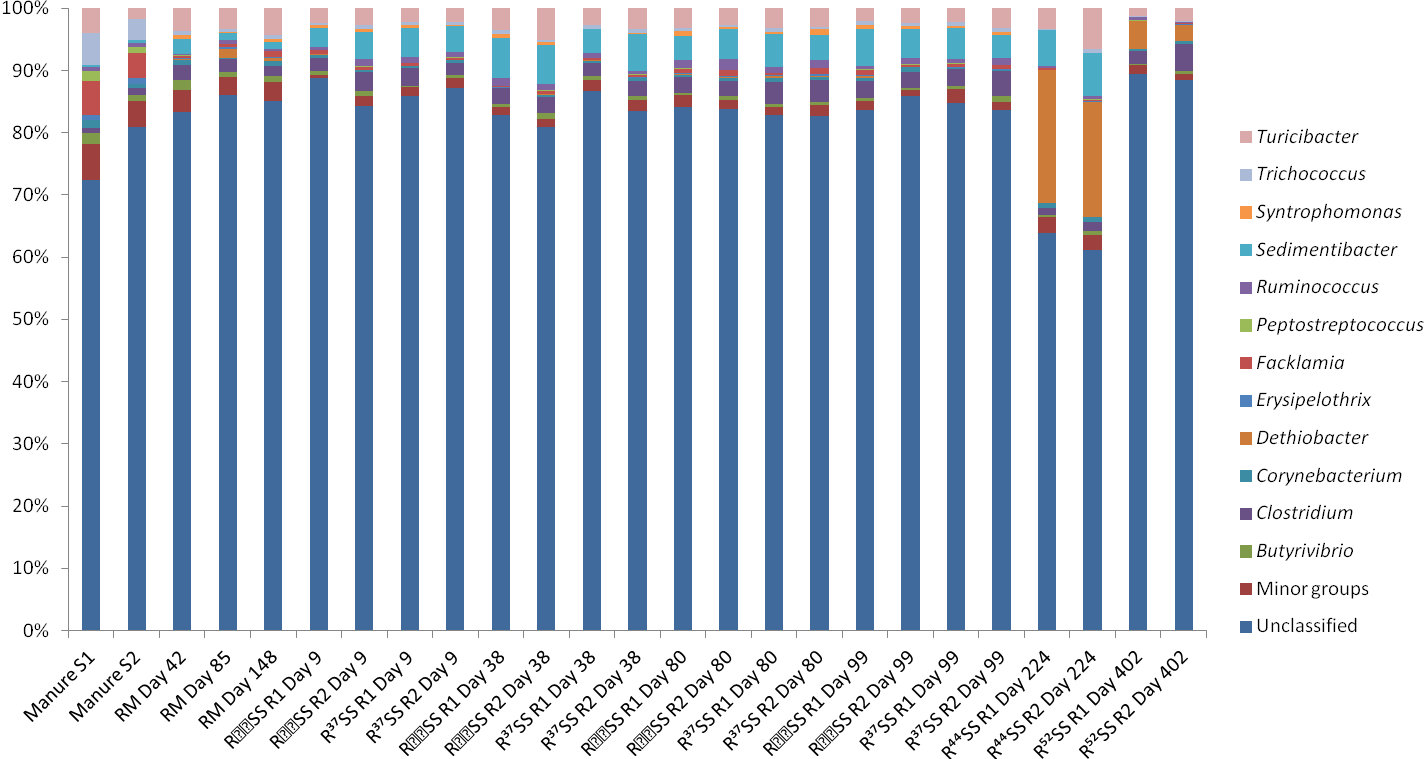


Figure S3. Relative abundance of bacterial 16S rRNA gene at phylum level in manure (S1 and S2 correspond to duplicate samples) and in laboratory-scale digesters processing: (1) manure as sole substrate (RM); (2) stream-exploded straw and manure, operating constantly at 37°C (R^Tc^SS); (3) steam-exploded straw and manure, operating temperature 37°C, 44°C and 52°C (R^37^SS, R^44^SS and R^52^SS), where R1 and R2 represent parallel digesters.


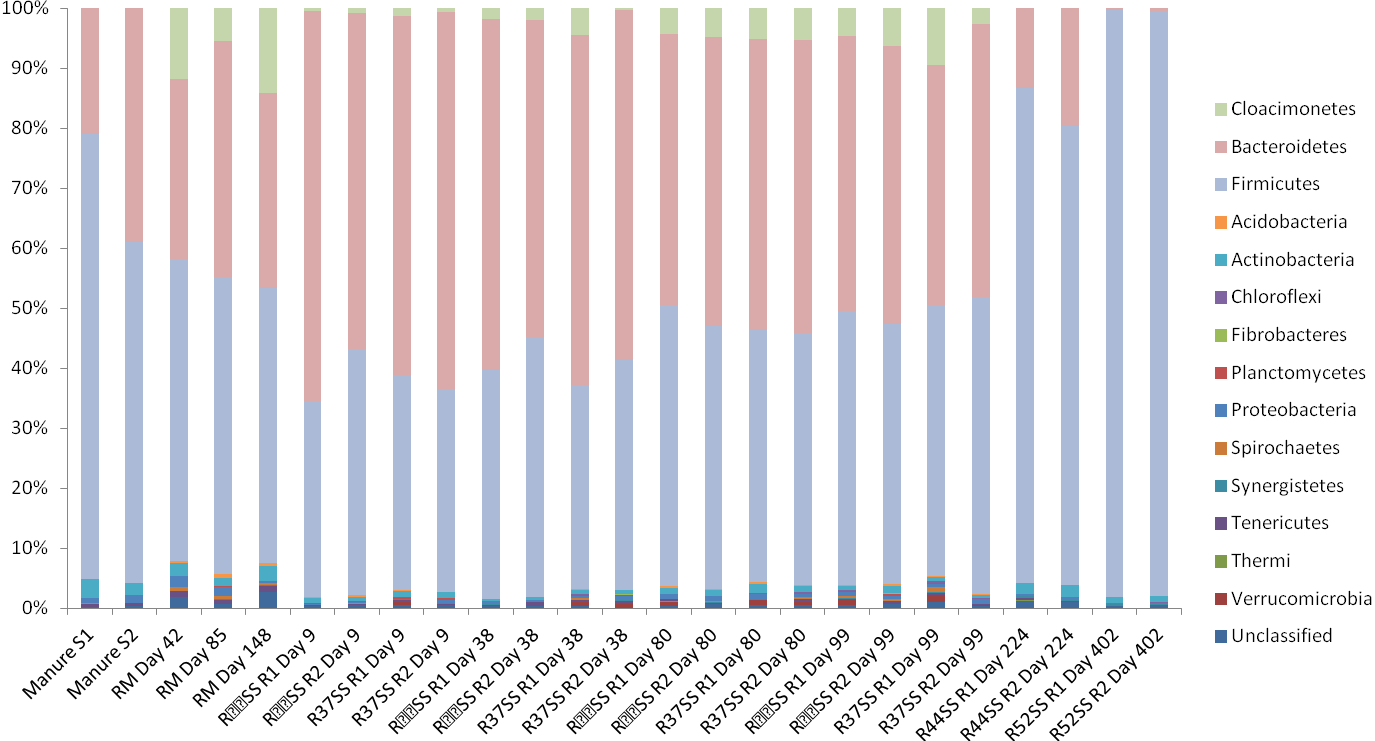

Supplement: Supplementary file 1 [file mbt20008-0815-sd1.docx]
